# Immunomodulatory Effects of Vitamin D in Respiratory Tract Infections and COVID-19 in Children

**DOI:** 10.3390/nu15153430

**Published:** 2023-08-02

**Authors:** Maria Nicolae, Cristina Maria Mihai, Tatiana Chisnoiu, Adriana Luminita Balasa, Corina Elena Frecus, Larisia Mihai, Vasile Valeriu Lupu, Irina Ion, Alexandru Cosmin Pantazi, Andreea Nelson Twakor, Antonio Andrusca, Claudia Simona Cambrea, Ioan Anton Arghir, Ancuta Lupu, Oana Cristina Arghir

**Affiliations:** 1 Department of Pediatrics, County Clinical Emergency Hospital of Constanta, 900591 Constanta, Romaniairynaion90@gmail.com (I.I.);; 2 Department of Pediatrics, Faculty of General Medicine, “Ovidius” University, 900470 Constanta, Romania; 3 Department of Pediatrics, “Grigore T. Popa” University of Medicine and Pharmacy, 700115 Iasi, Romania; 4 Faculty of General Medicine, “Ovidius” University, 900470 Constanta, Romania; 5 Department of Infectious Diseases, Faculty of General Medicine, “Ovidius” University, 900470 Constanta, Romania; 6 Department of Pneumophtisiology, Faculty of General Medicine, “Ovidius” University, 900470 Constanta, Romania

**Keywords:** vitamin D, COVID-19, pediatrics, respiratory tract infections, pneumonia, SARS-CoV-2

## Abstract

Acute respiratory tract infections (ARTIs) are one of the main reasons that the pediatric population goes to the doctor. The connection between ARTI and vitamin D (VD) is currently debated by the medical community, and so far, there has been little agreement with regard to the ideal level of 25(OH)D concentration that would provide protection for the respiratory tract, or the effectiveness of its administration in the treatment of respiratory infections. The purpose of this literature review was to bring attention to the immunomodulatory and antiviral function of vitamin D and its relation to the respiratory system by examining the main ARTIs, including SARS-CoV-2. The latter has affected the pediatric population in different ways, from asymptomatic patients to severe forms with multisystem inflammatory syndrome in children (MIS-C). Although there are not much clinical data on the SARS-CoV-2 disease in the pediatric population worldwide, we tried to find out whether there is a connection between the severity of this disease, other ARTIs, and vitamin D supplementation. We also aimed to find out if 25OHD deficiency had an adverse effect on the evolution of the disease and the recovery period in the case of younger patients affected by COVID-19. For this literature review, the PICO framework was selected as the methodological approach. Our results demonstrated many methods by which this vitamin may lower the risk of ARTI with regard to the COVID-19 infection. Despite these significant advancements, more research is needed to support the idea that 25(OH)D concentration can influence the evolution of respiratory tract infections in children.

## 1. Introduction

Worldwide, nearly 20% of all pediatric fatalities occur because of acute respiratory tract infections (ARTI) [1]. In accordance with 2020 Romanian statistical data from the INS (National Institute of Statistics), perinatal conditions (21%), and traumatic injuries (20%) overtook respiratory diseases as the third main reason for fatalities in young patients aged 0–19 years, accounting for up to 18% of 374 total deaths included in this group [2].

Upper respiratory tract infections (URTIs) and lower respiratory tract infections (LRTIs) are subsets of ARTI. A significant majority of URTIs are strongly related to viruses, with rhinovirus being the most prevalent one [3]. The SARS-associated coronavirus, adenovirus, enterovirus, influenza virus, and respiratory syncytial virus are among the other viruses [4]. Rhinovirus-related common cold cases surge in the autumn. Statistical analyses conducted in the US, for example, show that more than 40 million days of lost work and missed school are due to upper respiratory tract infections, creating a significant financial burden [5]. Pneumonia and bronchiolitis are the most prevalent LRIs in children [6], and statistics show that every 43 s, a child dies due to this LRI complication [7]. The same data indicators show that the total number of pneumonia-related fatalities among the pediatric population a day is over 2000, or over 700,000 children under the age of five, including over 200,000 newborns. The same indicators also state that for every 100,000 children worldwide, pneumonia affects more than 1400 of them, equating to 1 in 71 cases. The highest incidence figures are seen in South Asia (with 2500 patients in every 100,000 children) and Africa (West and Central), with 1620 occurrences per 100,000 children [7].

LRTIs are more common in children who lack vitamin D, which is a crucial component of immune function, which plays a role in modifying acquired immunity [8]. Thus, vitamin D stimulates innate immunity, decreases the proliferation of helper T lymphocytes (types 1, 17), and increases the production of type 2, together with regulatory T lymphocytes [9]. This, in addition to vitamin D, helps decrease the pro-inflammatory cytokines IL1, IL6, IL12, TNFα, IL17, and interferon γ. It also increases the number of anti-inflammatory cytokines IL10 [10] through the NFkβ metabolic pathway [11,12].

[25(OH)D] also exhibits antiviral action through antimicrobial peptides. Indeed, 25-hydroxycholecalciferol is converted to 1,25-dihydroxycholecalciferol in monocytes and macrophages expressing CYP27B1 (1-α hydroxylase). Moreover, the production of antigen cells is enabled by vitamin D, further influencing the role of innate immunity [13]. Calcitriol plays an important role in producing cathelicidin, β-defensin 2, and NOD2 proteins. Thus, it limits the presence of pathogens by destroying them. Nevertheless, vitamin D is responsible for the increased production of nitric oxide; thus, 25OHD exerts antioxidative effects [14,15].

With an increasing amount of research using cell-based experiments and preclinical models of illnesses, the possible extra-skeletal activities of vitamin D generated a great deal of interest in recent years. This is because both the vitamin D receptor (VDR) and CYP27B1 are found in a significant number of cells and tissues that are unrelated to the traditional target tissues for vitamin D [16]. Furthermore, vitamin D may play an extra-skeletal role through various mechanisms, such as epithelial integrity [17]. Gap and tight junction proteins, which support the maintenance of the integrity of epithelium, are expressed more rapidly when vitamin D is present [18]. Additionally, it has an indirect effect when it promotes self-eating, which leads to the death of virus-infected epithelial cells. The outcome described is achieved by modulating the metabolic pathway, mTOR [11].

### 1.1. COVID-19 and Vitamin D

In December 2019, the World Health Organization (WHO) declared a worldwide health pandemic caused by SARS-CoV-2 in response to the first virus outbreak reported in China, subsequently identified as COVID-19 disease [19]. From then on, several countries applied social distancing policies, stay-at-home policies, and they introduced lockdowns to reduce the spread of the illness after it was determined that the disease spread rapidly via a variety of routes, but primarily through the respiratory system [20]. It Is known that 25(OH)D concentrations are directly influenced by sunshine, and indoor behaviors, lockdowns, and other rules restricting movement to prevent the spread of COVID-19 impacted 25(OH)D concentration in children and adults [21]. Some of the clinical symptoms include high temperature, mucous, and cutaneous manifestations. Other symptoms such as rash, pink eye (conjunctivitis), hands/feet edema, and swollen and red tongue are also present at the time of disease onset [22]. With regard to children, multisystem inflammatory syndrome in children (MIS-C) is characterized by myocardial failure, complication of heart rhythm, as well as gastro-intestinal manifestations. The syndrome also puts younger patients at risk for lymphadenopathy and pulmonary problems [23].

As already mentioned, nuclear VDRs, which are primarily present in antigen-presenting cells (dendritic cells, macrophages, T cells, and B lymphocytes), are helping vitamin D in exerting its effects [24]. In the literature, vitamin D is described as a steroid hormone, which means it may pass through the lipid membranes straight to the nuclear receptor [25]. The genes that regulate the production proteins and inflammatory mediators necessary to change the phenotypes of immune cells are transcriptionally regulated because of this binding activity [26]. According to several studies, [25(OH)D]/VDR signaling pathway controls the renin–angiotensin–aldosterone system (RAAS), which modulates neutrophil activity, the pulmonary epithelial barrier, and epithelial repair [27,28,29,30]. Type II cells, monocyte/macrophages, and activated lymphocytes express the VDRs and enzymes of the vitamin D endocrine system. One way in which the VDRs influence the outcome of COVID-19 is through the production of calcitriol, as this decreases, directly and indirectly, the risk of pulmonary/systemic thrombosis, as well as hypercoagulability, by reducing the force of cytokine and chemokine storm. Additionally, VDRs modify the activity of neutrophils and preserve the pulmonary-epithelial barrier by increasing the capability of repair in case of lesions [31].

Different components of immunity may be modulated by (25[OH]D), which can change the severity of COVID-19 for the better [32]. Downregulation of ACE-2 receptors in the pathogenesis of COVID-19 results in toxic Angiotensin II buildup, a case in which acute respiratory distress syndrome (ARDS) is triggered [33]. Vitamin D helps interactions between SARS-CoV-2 and RAAS by stimulating ACE2/Ang-(1–7)/Mas receptor axis (having a vasodilation effect). As a negative endocrine regulator on RAAS, this protects against lung damage and ARDS [34]. ACE-2 receptor represents the gateway of COVID-19 in the cells; thus, vitamin D has a significant role in blocking the renin-production enzymes. It achieves this by attaching to the VDR, inhibiting the production of renin and, in such a way, limiting the entry of the virus into the cells [34].

### 1.2. Pneumonia and Vitamin D

According to WHO’s criteria, pneumonia represents cough or breathing difficulty together with at least one of the following symptoms: tachypnoea (2 to 11 months of age–50/min; age 1 to 5–40/min); lower chest wall pulling inward; crackles or pleural rub at chest auscultation [35]. If central cyanosis or oxygen saturation below 90% on pulse oximetry is present, together with severe chest indrawing, these symptoms meet the criteria for severe pneumonia [36].

Regarding ARTIs, 25(OH)D concentration is crucial. The literature shows that immunological deficiency and pathogen infection are factors in developing LRTI [37]. 25 Hydroxyvitamin D could contribute to acquiring pneumonia since vitamin D significantly impacts immunological processes [38]. Part of the physiology of vitamin D is the binding of VDR to the vitamin’s activated form 1, 25-(OH)2D3 [37]. With the stimulation of VDRs, the production of antibacterial peptides increases. Thus, bacterial and viral infections are limited [39]. The alteration of epithelia of the mucous membrane in the respiratory tract because of vitamin D deficiency or insufficiency (VDD or VDI) leads to a decrease in VDR levels, which damages the epithelial ability to face the increased production of inflammatory cells. This further damage the lungs and modifies the exchange of respiratory gases [40].

According to the US Centers for Disease Control and Prevention (CDC), neonates with low 25(OH)D concentration have a six times higher chance of developing lung infections when they reach the age of 1, when they are compared with infants with sufficient levels of the vitamin [41].

### 1.3. Bronchiolitis and Vitamin D

A severe risk in the morbidity of infants and young children is acute bronchiolitis. The respiratory syncytial virus (RSV) is the leading cause of this disease, especially in children under two years of age. The inflammation of the bronchioles causes extended expiration and wheezing; chest retractions are also part of the symptoms, along with tachypnea [28]. The significance of vitamin D is widely understood; it is crucial for the innate immune system’s activation, especially during lower respiratory tract infections. Defensins and cathelicidin, endogenic antimicrobial proteins that protect bronchial epithelial cells from bacterial and viral infections, are activated by vitamin D [42]. Since ultraviolet B ray exposure contributes significantly to influencing 25(OH)D concentration, its status is known to decrease during wintertime, when acute bronchiolitis is most common. In addition to their role in other immunological processes such as chemotaxis, cytokine release, inflammation, vascular permeability, and repair, cathelicidin is known to have antimicrobial action [43]. Additionally, vitamin D reduces the Th1 response by preventing the release of IL12, and it increases the Th2 response by directly triggering Th2 differentiation, primarily via IL4. By lowering Th1 lymphocytes’ release of pro-inflammatory cytokines and downregulating the formation of memory cells and plasma cells, vitamin D inhibits adaptive immunity [44].

However, the opinions on this are mixed. A study carried out by Biegelman et al. on 145 patients under one year of age concluded that there was no correlation between acute bronchiolitis severity markers and 25(OH)D concentration at the time of the disease, with only 9.7% (14) of the infants having VDD [45].

Another prospective multicenter cohort study analyzed the data for 144 infants aged 12 months or less. The young patients were hospitalized with bronchiolitis and they underwent metabolomic profiling of nasopharyngeal airway samples. They had a median 25OHD concentration of 23.7 ng/mL and a median free of 10.0 pg/mL, with a strong association between these values (r = 0.66). Research findings imply that circulating 25OHD affects the airway metabolites connected to inflammatory pathways, hence influencing the severity of the illness [46].

## 2. Materials and Methods

PubMed was searched for relevant articles using the following keywords “respiratory tract infections”, “pneumonia”, and “COVID-19” in combination with ”vitamin D” and “pediatric”. We also manually searched all eligible original articles, using the references of the first search results, reviews, and other relevant articles. No ethical approval was needed, as this study is a literature review.

The selection was limited to the following inclusion criteria: only free full texts in the English language were accepted; the type of articles searched were clinical trials, meta-analysis, RTCs, reviews, and systematic reviews; the population group included children from birth until 19 years of age only; and finally, the search criteria only included articles published in the last five years (May 2018–May 2023). Articles that could only be read in abstracts, posters, editorials, and comments were excluded from consideration.

The exclusion criteria involved: sample size (we looked only for articles with a sample size of at least 100 participants under 19); peer-reviewed literature (studies not peer-reviewed were not accepted). Case studies and articles targeting adults along with children were also excluded. Studies with incomplete data and those that could not provide quantifiable results for outcomes were also omitted.

The purpose of this literature review was to emphasize the immunomodulatory and antiviral function of 25(OH)D, and how it relates to the respiratory system, and although there are not much clinical data about SARS-CoV-2 infection in children worldwide, we tried to find out whether there is a connection between the severity of this infection and other ARTIs with the VD supplementation. Nevertheless, SARS-CoV-2 has affected the pediatric population differently, from asymptomatic patients to severe forms with MIS-C. We also sought to find out if the 25OHD deficiency had an inverse relationship with the clinical condition, the evolution of the disease, or the recovery period of these young patients.

A systematic review as a methodological approach using the framework of Patient, Intervention, Comparison, and Outcome (PICO) was used as a tool for research.

Population: pediatric population with acute respiratory disease: acute pneumonia, bronchiolitis, recurrent wheezing, seasonal infections with the Influenza A virus, COVID-19.

Intervention: Vitamin D—oral or injectable administration, in a single dose per day/week or less often, in doses varying from 400 UI/day to 2000 UI/day or 14,000 UI/week. The duration of administration varied from a few weeks to 8 months.

Comparison: Standard care and placebo.

Outcomes: To highlight the immunomodulatory effects of vitamin D in ARTIs (including COVID-19) in children.

Preferred Reporting Items for Systematic Review and Meta-Analysis (PRISMA) were used to report the review.

After careful selection, the publications that looked at the 25(OH)D effect on different respiratory infections in study participants or in placebo groups were selected for further analysis. The relationship between vitamin D and these diseases was classified into two sets: positive associations (marked with “YES”) and negative associations (marked with “NO”). “YES” associations meant that there was a proven relationship between increased VD levels and a more favorable respiratory infection result. Studies were only included if they had outcomes that could be quantified, and the full text was available.

**Figure 1:**
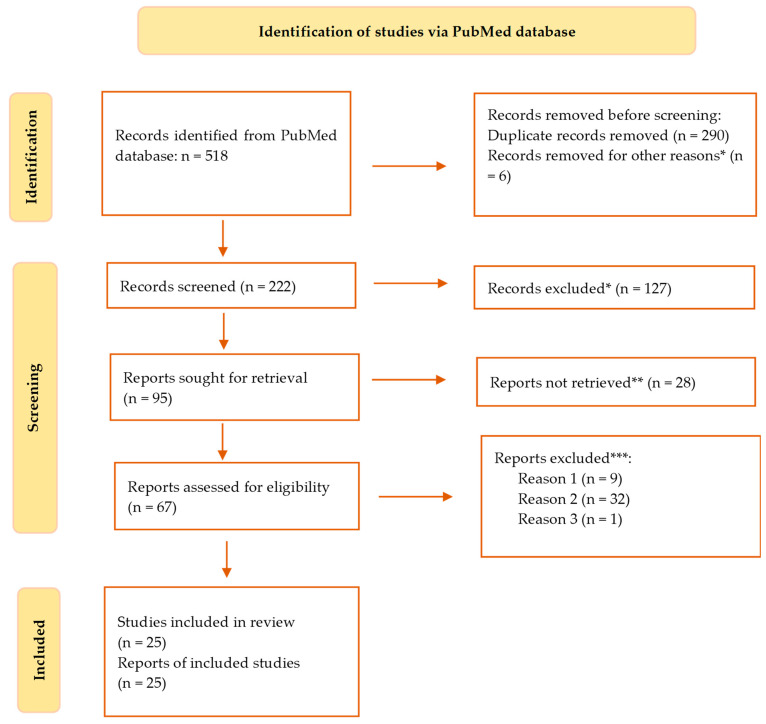
Flowchart of the course of selection of documents in the systematic review of the literature following the PRISMA model. * studies are not relevant for the present review; ** studies do not help us to provide an answer to the research question; *** unable to find the full text of the study; *** Reason 1—wrong setting; reason 2—wrong patient population; reason 3—research question not relevant.

A total of 518 citations were found after searching the PubMed database (Figure 1). After removing duplicates and six other articles that did not meet the search criteria and appeared erroneously in the results, 228 were still on the list. Out of these, 127 studies were disregarded because, based on their abstracts, it was evident that they did not fit the requirements of our research, 28 papers were further dropped from consideration because they did not answer the question of this study, 9 more were excluded because access to the complete text was impossible, 32 were also omitted due to the wrong age group, 1 article was ignored as it was written in a different language than English. At this point, we had 27 search results that were eligible for our study.

Thus, the final literature review contained 25 papers that satisfied the inclusion requirements. We systematized the data from these articles in Table 1 and Table 2, which is displayed in the Section 3.

Below is a breakdown of the results from the PubMed search, including the filters applied when we carried out the search:

Search results for “respiratory tract infections and vitamin D”—217 results;
Search results for “vitamin D pneumonia”—134 results;
Search results for ”vitamin D and COVID-19”—104 results;
Search results for ”vitamin D and SARS-CoV-2”—63 results.

The filters applied in our search on PubMed were: Free full text, Clinical Trial, Meta-Analysis, RCTs, Review, Systematic Review, English, Child: birth-18 years, Newborn: birth-1 month, Infant: birth-23 months, Infant: 1–23 months, Preschool Child: 2–5 years, Child: 6–12 years, Adolescent: 13–18 years, in the last 5 years (May 2018–May 2023).

## 3. Results

Table 1 shows the outcomes of the RCTs and prospective cohort reports in this literature review.

Table 2 describes the results of the meta-analyses included in this study.

**Figure 2:**
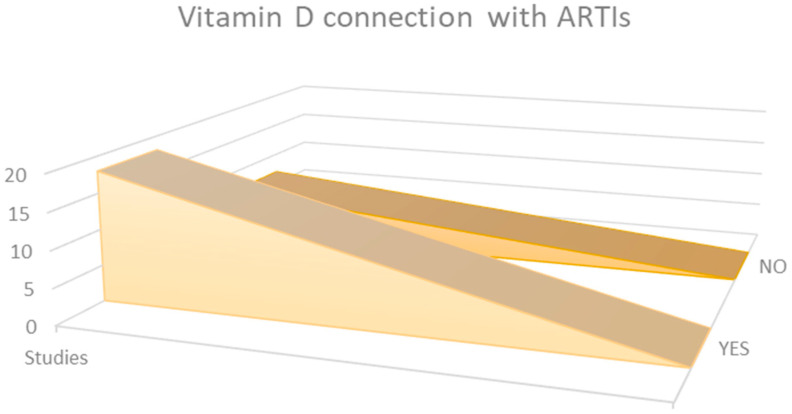
Comparison of results from the 25 selected studies.

Observational data from the 25 research studies reviewed in this analysis indicated that quantities of [25(OH)D] are, in most cases, associated with the rate, outcome, and, nevertheless, mortality percentage when it comes to ARTIs (Figure 2).

The studies of Chowdhury et al., Loeb et al., Hueniken et al., and the two conducted by Oktaria et al. showed that there are insufficient solid data to recommend a regular megadose vitamin D3 supplement for young children with severe ARTI, either for treatment or prevention [48,51,58,60,61]. Moreover, a high dose of vitamin D supplementation did not reduce RTI symptom severity, outpatient visits, ED visits, or antibiotic prescriptions.

The work of Zhou et al., Chowdhury et al., Alpcan et al., and Vo et al. demonstrates a total reduction in time to recovery from pneumonia and other respiratory infections, as well as the overall duration of hospital [47,48,50,56]. According to these studies, high-dose vitamin D (1200 IU) is suitable for preventing seasonal RTIs, as evidenced by rapid relief from symptoms, a rapid decrease in viral loads, and disease recovery. Labib et al., Cariolou et al., Kuang et al., and Alakaş et al. provided evidence that the supplementation of vitamin D lowers the risk of ARTI evolution and death [49,53,55,66]. In these studies, VDD was detected in children who were in a critical state. In those having RTIs coupled with high VDD, the authors illustrated that vitamin D supplementation is accompanied by lowered mortality risk and qSOFA scores and reduced recovery time.

Furthermore, a strong association between 25(OH)D concentration and the severity of ARTIs was proved by Pham et al., Loeb et al., Zhang et al., Golan-Tripto et al., Kun et al., and Oktaria et al. [51,52,54,59,60,61,68]. Their research revealed an association between VDD and clinical severity, and inflammation markers in pediatric ARTIs cases. Also, the risk for intensive care during hospitalization was shown in the studies conducted by Toivonen et al., Oktaria et al., and Akeredolu et al. [57,60,61,62]. Here, there was a positive relation between vitamin D concentration and the risk of needing intensive care.

In addition to this, Bayramoğlu et al., Doğan et al., Yılmaz et al., and Hong et al. showed that supplementation of vitamin D could be used as a supportive treatment in ARTIs and food fortification with vitamin D should be strongly considered as a long-term strategy [63,64,67,72].

### Statistics of the Main Findings

**Figure 3:**
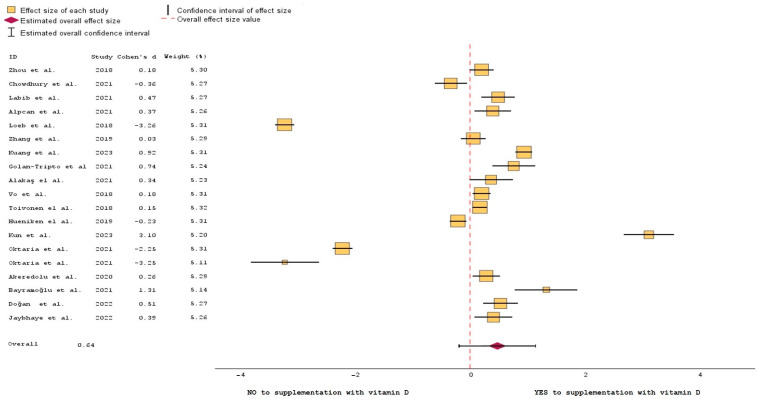
Forest plot of the efficiency of vitamin D supplementation on the outcome of ARTIs [47,48,49,50,51,52,53,54,55,56,57,58,59,60,61,62,63,64,65].

**Figure 4:**
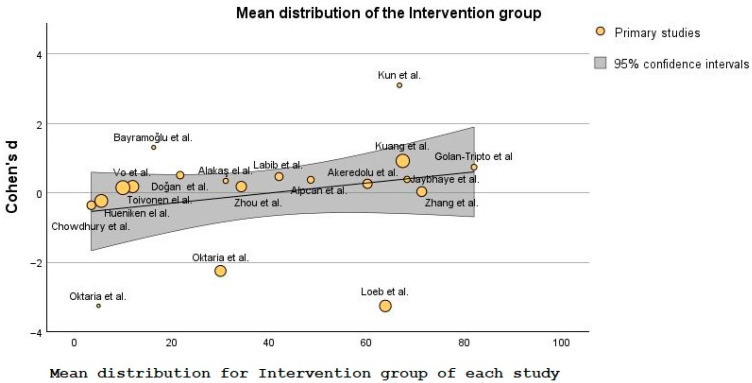
Bubble plot showing the differences in SD between intervention groups [47,48,49,50,51,52,53,54,55,56,57,58,59,60,61,62,63,64,65].

**Figure 5:**
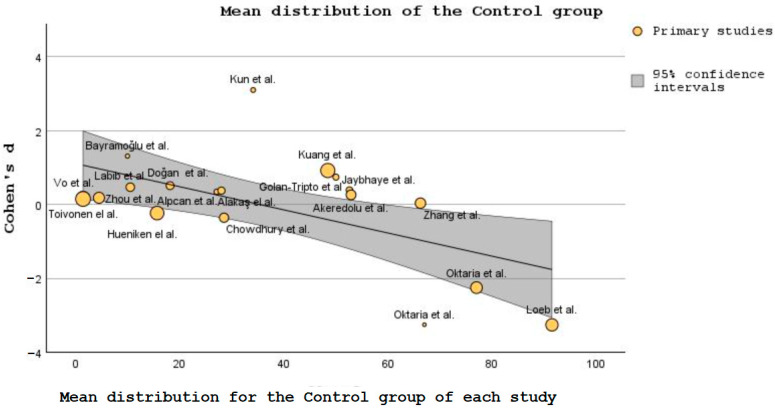
Bubble plot showing the differences in SD between control groups [47,48,49,50,51,52,53,54,55,56,57,58,59,60,61,62,63,64,65].

The data generated from the studies listed in Table 1 helped create a forest plot (Figure 3) and a bubble plot (Figure 4 and Figure 5).

Figure 3 compares the effects of vitamin D supplementation on changes in the outcome of ARTIs using random effects meta-analysis. The forest plot shows that the vitamin D intervention was more effective than the baseline regarding absolute change in [25(OH)D]. Overall, using a random-effects model, supplementing with vitamin D had a higher impact on the favorable outcome of ARTIs than not supplementing. In a forest plot, each study is represented by a line or a square, with the horizontal line indicating the confidence interval for the study’s effect size estimate [73]. The vertical axis lists the individual studies. The effect size estimates are displayed as squares (some bigger than others, depending on the weight of the study), positioned along the horizontal line at the point estimate of the effect size [74]. The length of the horizontal line represents the confidence interval for the effect size estimate, with longer lines indicating greater ambiguity [75].

Cohen’s d is a measure of effect size that quantifies the standardized difference between the studies that concluded either NO or YES to vitamin D supplementation.

Studies conducted by Chowdhury et al., Loeb et al., Kun et al., and the two studies conducted by Oktaria et al. had the greatest uncertainty when it comes to the results of their trials [48,51,59,60,61]. Thus, they are represented graphically in the left side of the plot. The summary estimate, represented above by a diamond-shaped marker, provides an overall measure of the effect size based on the combined evidence from all the included studies. In this case, the diamond is positioned to the right, in favor of vitamin D supplementation, which is the combined effect of all the 19 studies included in the analysis (five for NO supplementation, fourteen for YES supplementation).

Figure 4 and Figure 5 contain bubble plots, which is a type of data visualization that displays three variables simultaneously on a two-dimensional graph [76]. The three variables included in these plots are: Cohen’s d, patient’s mean distribution in the intervention/control groups, and the additional variable is represented by the size of the bubbles plotted on the graph. Each’s bubble position in the chart represents where that study is in terms of mean distribution when compared with the others, and the bubble size represents the sample size of each of the 19 intervention or control groups, respectively. It could be argued here that the studies with bigger bubbles carry more weight as the number patients is wider and it covers more of the population sample.

Cohen’s d indicates the magnitude of the difference between the groups in standard deviation units [77]. In Figure 5, Cohen’s d shows that for the intervention or control groups, several studies greatly differed from the ones inside the bubble [59,60,61,63].

Concerning Figure 4 and Figure 5, the variability of the means between studies was used to quantify the strength and direction of the relationship between these variables [78].

## 4. Discussion

The articles in this review demonstrate a connection between VD status and the occurrence and outcome of ARTIs, including COVID-19 infections. Two further systematic reviews [79,80] found the same results. Although Pereira et al.’s meta-analysis found no correlation between VDD and a higher prevalence of COVID-19 infection, most observational studies that were included in his analysis found an inverse correlation between vitamin D levels and the severity of COVID-19 [79]. If further research into the effects of vitamin D will reach similar conclusions, then major public health problems can be addressed. So far, studies that were researched here indicated an association between vitamin D and the COVID-19 mortality rate. These also point to significant possibilities for health promotion in terms of preventing vitamin D insufficiency across the globe [81,82].

Reverse causality is an instance in which the result comes first, and then the exposure, is a significant bias that is sometimes present in medical research that attempts to link a risk factor to a sickness (ARTIs in this review) [83].

Since some of the young patients from the RCTs included in this literature review were hospitalized when the vitamin D concentrations were determined, those specific studies did not include baseline measurements. As a result, based on these facts, it was difficult to confirm the conclusion of causality.

We came across an instance of reverse causality with the expression CYP24A1, which is increased in COVID-19 infection [38]. Renal proximal tubular cells primarily express these genes involved in vitamin D production—CYP27B1, and its catabolic enzymes—CYP24A1 (both control the concentrations of 25(OH)D) [84]. Thus, vitamin D inefficiency may be a risk factor. However, this connection might be brought about by confusing or reverse causality, both COVID-19 and a lack of vitamin D are separately linked with outdoor exposure, age (over 65), and body mass index [85].

The interest in vitamin D’s properties intensified during the COVID-19 pandemic [86] since the relationship with the illnesses was already shown in studies such as the one conducted by Bergman et al. [87] Here, the authors demonstrated that 25 hydroxyvitamin D protects against ARTIs [87] the results of this paper revealed the same findings of a similar analysis in which vitamin D has a prospective protective and preventative impact when it comes to COVID-19 [88]

Moreover, recent investigations provided evidence that supports further reverse causation interpretation. Some studies included in this review indicated that an increase in inflammatory markers is linked to a decline in vitamin D serum concentrations [47,48,49,50,53,55,56,58]. However, this can be argued, as ARTIs often require LPS infusion, which, in turn, leads to an inhibition of vitamin D production; thus, a drop in 25OHD can be anticipated (therefore, not due to the disease itself) [89].

Poor vitamin D status has been linked in the literature to increased susceptibility to viral infections, especially RTIs [66,90]. Numerous variables are responsible for vitamin D’s ability to prevent ARTIs. 25 hydroxyvitamin D is thought to boost the synthesis of natural antibodies [41]. By causing monocyte differentiation and preventing lymphocyte proliferation, vitamin D is also known to boost immunity [43]. Additionally, other authors suggested that vitamin D increases macrophages’ phagocytic activity [91]. This review also showed that VD intake could minimize the risk of COVID-19’s severity. However, we believe that its benefits should be analyzed in a comprehensive clinical study, since 25(OH)D is generally safe, and a considerable body of evidence supports the use of greater doses of vitamin D3 in vitamin D deficiency [49,50,63,79,92].

A meta-analysis that summarized randomized controlled trials (RTCs) totaling 4786 children analyzed the efficacy of vitamin D supplementation and a combination of antibiotics to treat pneumonia [42]. Here, Yang et al. concluded that by boosting immunological effectiveness, a higher concentration of vitamin D interventions may lower the prevalence of recurring pneumonia [42]. Akeredolu et al. analyzed the vitamin D levels of 270 children aged 1 to 59 months, out of which 135 were part of the control group, and 135 were diagnosed with different levels of pneumonia. In this study, almost 67% were patients with low and moderate pneumonia, while over 33% were hospitalized patients with severe [62]. The average blood serum levels of vitamin D in the intervention group were significantly lower than that of the control group. The authors concluded that the 25(OH)D concentration higher than 75.0 nmol/L significantly reduced the odds of acute pneumonia, with 71.9% compared to 91.1% in the pneumonia group with [25(OH)D] concentration ≤75.0 nmol/L [62].

Another research that investigated the relationship between vitamin D status at admission and the severity of the illness in babies with bronchiolitis who are being treated in hospitals had similar findings [93]. 25(OH)D concentrations of 156 neonates were analyzed, and around 27% had VDI concentrations of less than 50 nmol/L. A further 27% of children were VDD with levels between 50 and 74 nmol/L, and only less than half had adequate levels of 75 nmol/L. Newborns who later developed bronchiolitis had 25-OHD concentrations of 65 nmol/L (VDD) vs. neonates who did not have the disease and that were VD sufficient. Compared to newborns with 25-OHD concentrations at 75 nmol/L or more, those with 50 nmol/L had a higher risk (six times) of developing LRTI in the first year of life [93].

Toivonen et al. identified that the most common reason for hospitalization of newborns in the United States is bronchiolitis, and 15% of them need intensive care [57]. The authors of this multicenter prospective cohort study found a significant risk of intensive care during bronchiolitis hospitalization in 1016 infants under 12 months with lower 25OHD concentration [57]. Admission to the ICU or the use of MV (CPAP or intubation) during a bronchiolitis hospital stay were considered the main outcomes of this study. In young patients admitted for bronchiolitis, nasopharyngeal microbiota profiles and blood total 25-hydroxyvitamin D status have been linked to risks of needing intensive care unit [57].

Manaseki-Holland et al. showed, in an RCT, that giving babies under 3 years old diagnosed with pneumonia 100,000 IU of VD3 almost eliminated the risk of recurrence within 3 months [94]. In another RCT from our analysis involving 133 children under 5 years of age, Oktaria et al. showed that 1/5 of children hospitalized with pneumonia were VDD [60]. Labib et al. analyzed 93 children with pneumonia who were also given 100,000 IU of VD3, while 98 received the placebo [49]. Auscultation results, temperature, respiratory rate, chest indrawing, SpO_2_, and nutritional status were all tracked and documented for all children every 8 h. When the effects of VD supplementation were assessed 7 days later, the experimental group had significantly higher levels of 25(OH)D concentration and a bigger fraction change than the placebo group on the last day of the intervention. Additionally, the median recovery time for the placebo group was much greater than it was for the vitamin D supplement group [49].

As a consensus, vitamin D insufficiency is defined as a 25-hydroxyvitamin D level below 21–29 ng/mL (50–75 nmol/L), while vitamin D deficiency is indicated by a level <20 ng/mL (<50 nmol/L) [62]. Severe vitamin D deficiency at <10–12 ng/mL (<25–30 nmol/L) is associated with the risk of osteomalacia in adults and rickets in children. Finally, 30–50 ng/mL (75–125 nmol/L) indicates sufficient vitamin D and is considered the normal range of 25-hydroxyvitamin D levels with a role in preventing both vitamin D deficiency and vitamin D toxicity [95].

In Table 3, we present the US recommended intake of VD.

The Australian government recommends that each day, people under the age of 19 and adults over the age of 50 should consume 5 mcg of vitamin D. Adults 51 to 70 years old should consume 10 mcg, and those over the age of 70 should consume 15 mcg of vitamin D daily [97].

The UK National Health Service advises that 10 mcg of vitamin D per day are required for adults and children over 1. This includes those who are at risk for vitamin D insufficiency as well as pregnant and nursing mothers. According to the NHS, 8.5 to 10 mcg per day are necessary for infants up to 1 year old [98].

The 2022 Central and Eastern European expert Consensus Statement recommendation also included a target level of 75 to 125 nmol/L for vulnerable categories of people, such as those risking fractures, falling over, autoimmune diseases, and malignancies [42]. In 2017, the Endocrine Society also advised increasing VD to 75 nmol/L or higher, for chronic patients [99].

A meta-analysis conducted by Jolliffe et al. agreed that protection against ARTI is indeed achieved by daily doses of 400–1000 IU for up to 12 months [97].

Protection can also be acquired by exposing arms/legs to sunlight for 30 min per day at an angle of 45°, this being equivalent to 3000 IU of vitamin D. Body exposure (swimsuit, no UV protection cream) is equal to 20,000 IU [100].

### Limitations

This research contained a few limitations. Firstly, the data may be inaccurate or partial because of the retrospective research design. Second, specific selection criteria, such as some clinical studies, might show results from geographical regions where children did not show low values of vitamin D by default (warm climate regions). Therefore, the overall conclusion might be affected by this. Also, we believe that the sample size of at least 100 participants under the age of 19 may be modest, but despite all these drawbacks, it might provide useful information for future research on the validity of a link between vitamin D insufficiency and COVID-19.

## 5. Conclusions

VDD and VDI are both significant problems across pediatric population. Several observational studies clearly linked low concentrations of 25(OH)D to an elevated risk of developing an ARTI. However, despite a few well-researched systematic reviews and meta-analyses, the experimental data on the effects of vitamin D on acute respiratory tract infection are mixed, making it impossible to advise the public to take vitamin D supplements as a preventative measure for these diseases. To reach this conclusion, we considered all the available scientific information to emphasize the importance of vitamin D for public health and provide advice on this matter.

Globally, widespread VDD has been miscalculated, and there is growing consensus that serum VD should be maintained above 75 nmol/L. Adding nutritional supplements to people’s diets would be an affordable method to improve the population’s overall health, particularly for the elderly and those who do not have access to enough direct sunlight, as numerous diseases are linked to VDI or VDD.

The result of our research complements the existing literature on the appropriate intake of this vitamin and its link to clinical outcomes in young patients with ARTIs. While it is not generally accepted that vitamin D contributes to the physiopathology of respiratory infections, further research is required to determine if there is, in fact, a link between vitamin D insufficiency and these diseases.

To maintain adequate amounts of vitamin D, we recommend giving vitamin D supplements to children and adolescents. Additionally, to avoid VDI in all age groups, dietary vitamin D intake (fish, fortified food) should be maintained, considering the pandemic’s ongoing impact on time spent outside and, therefore, sun exposure. By offering these results to the scientific community concerning vitamin D, we hope that our work contributes to the body of material already in existence.

## Figures and Tables

**Table 1:** Results of the 19 studies selected for this review with regard to the effect of vitamin D on various ARTIs.

Study	Study Design	Pico Framework	Results of the Study	Conclusion	VIT. D Supplementation
Zhou et al. [47]	RCT	Participants: In total, 169 children were included in the low-dose vitamin D group and 164 in the high-dose group (aged 3–12 months old) Intervention: Infants were randomly assigned to low-dose or high-dose vitamin D3 (VD3) groups, with children in each group receiving drops orally for 4 months (one drop containing 400 IU of vitamin D) Comparison: The two groups were compared for the suitability of administering vitamin D to prevent the development of seasonal influenza. Outcome: To assess vitamin D’s safety and clinical effectiveness in preventing influenza A.	In total, 78 (46%) and 43 (26%) influenza A infections occurred in the groups receiving low and high vitamin D doses, respectively. When comparing the two sets of children, there was a significant difference when it comes to the median durations of fever (22.18 ∓ 36.77 vs. 35.20 ∓ 47.53), cough (1.42∓2.57 vs. 3.43 ∓ 4.38), and wheeze (0.99 ∓ 1.82 vs. 2.83 ∓ 3.69) in the high dose and the low dose group, respectively.	A dose of 1200 IU 25(OH)D is suitable for lowering the risk of Influenza A. This conclusion is backed up by the rapid rate of disease improvement, lower viremia, and fast recovery. In addition, an increased dose of VD is probably safe for patients in this age group.	YES
Chowdhury et al. [48]	RCT	Participants: In total, 97 vs. 100 children included in the experimental and the placebo group, respectively (2–59 months old with severe pneumonia). Intervention: Depending on the group, children received either 20,000 IU per mL of vitamin D3 (a high dose or miglyol oil 812. Children could not tell the difference between these two substances, as they looked and tasted the same. Comparison: Children received a vitamin D3 doze according to their age, as follows: 20,000 IU for those under 6 months, 50,000 IU for children 6 to 12 months, 100,000 IU for the remaining ones 13 to 59 months old, and those in the placebo group received miglyol oil. Upon receiving the mega dose, all children included in the study received 10,000 IU doses for 4 days or until discharge from the hospital.Outcome: To evaluate the necessity of 25(OH)D3 supplementation in children admitted to the hospital with severe cases of pneumonia.	On admission to the hospital, the serum VD level was measured, and around half of the children from each group were VDD (52% the intervention group and 49% in the placebo group). Children with severe pneumonia from the 1st group seemed to recover faster (72 h pneumonia resolution time) when compared with the control group (88 h resolution time). The same trend was also noticed regarding hypoxemia, as children in the VD group were hypoxic around 24 h, while those in the 2nd group were hypoxic 48 h. A small difference was also noticed in the period needed for the full recovery of children. The VD group needed around 4 days, while the placebo group needed 5 days.	Even though there were some differences between the two groups, the authors concluded that the time needed for improving tachypnea, chest in-drawing, or the overall resolution of severe pneumonia, irrespective of VD status, were not that great. Also, not-so-great differences were registered regarding the number of days until hospital discharge of children from the two groups. A maintenance dosage of VD after an age-specific megadose was shown to not statistically vary from the other two intervention groups.	NO
Labib et al. [49]	RCT	Participants: In total, 191 children (1 month to 12 years old) were put into two groups, 93 in the experimental group and 98 in the control group. All children were either with VDI or VDD and were diagnosed with pneumonia. Intervention: A dose of 100,000 IU of VD3 (intravenous), or placebo. Comparison: To ensure that every participant was evaluated before being discharged, children were assessed for seven days following vitamin D treatment, especially on the day when vitamin D concentration in the blood reached their peak. Outcome: To evaluate the development and recovery time of the children.	Seven days after the injection with the high dose of VD3, the experimental group showed higher serum concentration levels. In this group, the percentage of patients who were VDI or VDD on the 1st day had significantly reduced by the last day (with 11.8% on the 1st day and 52.7% on the 7th day). Also, most of the children in the intervention group (93.5%) of the supplementation group registered normal PaO_2_/FiO_2_ values, from 300 to 360 (day 1 and day 7), compared with 340 to 345 (day 1 and day 7) in the intervention group and placebo group, respectively.	It was shown that a high dose of VD administration is indeed related to a decreased risk of mortality, shortened recovery time, better PaO_2_/FiO_2_, and lower qSOFA scores in pneumonic children with high VDD. Seven days following the intervention, most of the vitamin D group’s PaO_2_/FiO_2_ readings returned to normal, showing a reduction in the severity of lung damage in the children receiving mechanical ventilation (MV).	YES
Alpcan et al. [50]	RCT	Participants: In total, 155 pediatric patients (75 in the intervention group and 80 in the control group) with mild or moderate COVID-19 disease (1 month to 17 years old). Intervention: To determine vitamin D serum level and other biological analyses. Comparison: To compare the blood results between the groups. Outcome: To compare the VD levels of pediatric patients identified with COVID-19.	The average blood level of 25(OH)D concentration was lower in the 1st group than the control group (21.5 vs. 28.0), and the percentage of VDD patients was higher in the COVID-19 group than 2nd group (44% vs. 17.5%). The mean C-reactive protein (CRP) level was higher in the vitamin D-deficient children (9.6 vs. 4.5). Still the hospitalization days were similar for both groups, with a statistical difference of only r = 0.724.	Parallel examination of different blood results showed an essential relation between VD level and leukocyte, lymphocyte, and thrombocyte count and no negative association with age and duration of hospitalization. The regression analysis suggests an inverse relation between low vitamin D concentration and the risk of developing respiratory infections.	YES
Loeb et al. [51]		Participants: Two groups totaling 1300 healthy children (3 to 17 years of age) were divided between two groups, each with 650 children (vitamin D and placebo). Intervention: Those included in the study were randomized, half of them receiving 7 drops of vitamin D (0.028 mL per drop—14,000 U/week) every week for 8 months, while those arbitrarily receiving were administered 7 placebo drops (0.028 mL per drop) for the same period. Comparison: Children in the two groups were compared for signs and symptoms of influenza two times every week over a period of one year. Outcome: To analyze RT-PCR-confirmed infection with influenza and PCR-confirmed non-influenza respiratory viruses.	In total, 7.7% (50) of children in the intervention group were confirmed by RT-PCR with influenza A or B, while only 1% less (6.6%, or 43 children) were RT-PCR confirmed with the same virus. Additionally, regarding other viruses, 146 patients (22.5%) from the VD group acquired non-influenza infections, compared with 185 patients (28.5%) in the placebo group. Thus, 25 hydroxyvitamin D’s had some effect in reducing all respiratory infections, 177 of the total 650 (27.2%) in the VD group compared with 209 in the 2nd group (32.2%).	The conclusions of this study suggest that 25 hydroxy cholecalciferol supplementations did not reduce the incidence of influenza A or B virus but reduced in a small percentage the other respiratory infections. Therefore, it can be presumed that VD supplements can have some influence in reducing viral respiratory infections. Still the authors conclude that it is not enough to recommend the routine administration of it to the general pediatric population.	NO
Zhang et al. [52]	RCT	Participants: In total, 422 youngsters were included in the intervention group and 100 in the control group were observed for recurrent respiratory infections (RRIs). Intervention: Serum levels of 25 (OH) D2, and vitamin A, and E were analyzed. Comparison: The study group was further separated into two subgroups, with an active group of 222 children, and a stable group of 200, along with other 100 healthy children and they were all compared for their risk of developing RRIs. Outcome: To analyze if there is any connection between the different vitamins (A, D and E) and RRIs among the pediatric population.	The study found that the relationship between vitamins A and D was positive in children with RRIs (r = 0.945 for vit. A and r = 0.988 for vit. D). The relationship between vitamin E and vitamin D was also positive in those children, with r = 0.959 for vitamin E (vitamin D having an r = 0.988).	Deficiency in the three vitamins relates to RRIs in children. Thus, supplementing vitamins A, D, and E via dietary changes is advantageous for the faster recovery of the patients.	YES
Kuang et al. [53]	RCT	Participants: In total, 948 children aged 0 to 16 years, divided as follows: 295 with ARTIs, 17 with co-infection and 636 healthy children. Intervention: PCR or (RT)-PCR was used to examine oropharyngeal samples from patients, detecting the existence of viruses or unusual infections. Comparison: The study compared the results of the PCR and RT-PCR of the intervention group and the healthy group. Outcome: To determine if there is a connection between the vitamin D status in children with ARTI due to viruses or other unusual pathogens.	Of the total 295 children infected with a single virus, more than half (58.98%) were VDD (>50.0 nmol/L), together with a large percentage of the 17 children infected with more than one virus (76.47%). However, regarding the ARTIs and the co-infected sub-groups, the differences in the VD levels were not great.	The study showed that VDD is associated with developing ARTIs with typical or atypical pathogens. Thus, the recovery of ARTIs may be correlated with serum 25(OH)D concentrations.	YES
Golan-Tripto et al. [54]	RCT	Participants: In total, 127 patients less than 24 months were divided into an acute bronchiolitis group (80 children) and a group of 47 children with other non-respiratory illnesses. Intervention: Blood samples were collected to determine if the children were either VDD (<50 nmol/L), VDI (≥50 and <75 nmol/L), or sufficient (≥75 nmol/L). Comparison: The vitamin D status of the children was compared against the severity of bronchiolitis and length of hospital stay. Outcome: To analyze if there is a connection between the 25(OH)D concentration in very young patients diagnosed with acute bronchiolitis compared to other infections not related to respiratory tract.	In this study, more than half of the children from each group received vitamin D supplementation (66% and 60% in the bronchiolitis group and control groups, respectively). In the bronchiolitis group, blood 25(OH)D concentration was considerably lower when compared with the control group. The statistics showed a median of 28 vs. 50 nmol/L, respectively. VDI happened more likely in the bronchiolitis group, according to the regression model used by the authors.	Even though the authors did not observe any association between 25(OH)D concentrations and bronchiolitis severity, they noticed that children in acute bronchiolitis group displayed lower vitamin D levels than children from the 2nd group. Also, they required a longer recovery period, measured in the length of days spent in the hospital.	YES
Alakaş et al. [55]	RCT	Participants: In total, 182 children with acute bronchiolitis between 1 month and 2 years of age were divided into a group of 73 children (40%) with severe cases of disease admitted in the ICU department, and 109 children (60%) in the typical patient ward. Intervention: An amount of 2 mL of blood was collected for vitamin D examination. This was done on the same day as admission to the hospital, along with other regular blood analyses. Comparison: The vitamin D concentration of those admitted in the ICU department as opposed to those that did not need intensive care. Outcome: To assess if VD is linked to the symptoms and outcome of acute bronchiolitis in very young patients.	When comparing the progress to the ICU department, the study shows that 25 (34.2%) of children whose VD levels were normal (73), 29 (39.7%) who were VDD, and 19 (26%) who were VDI needed intensive care. The trend is similar with the 109 patients admitted to the normal patient ward. Therefore, 70 (64.2%) children had sufficient VD concentration, 25 (22.9%) and 14 (12.8%) were VDD and VDI, respectively.	A significantly higher likelihood of VDD or VDI was found in patients diagnosed with bronchiolitis that needed intensive care compared to those in normal patient wards. Hence, vitamin D deficiency is indeed correlated with severe bronchiolitis.	YES
Vo et al. [56]	Prospective cohort study	Participants: In total, 1016 infants under 12 months hospitalized with bronchiolitis were divided into 3 groups, depending on blood 25(OH)D levels: 298 children (<20 ng/mL), 352 with vit. D values of 20–29.9 ng/mL, and 366 children having serum concentrations of ≥30 ng/mL. Intervention: Blood was collected within 24 h of patients being admitted to the hospital. Using a standardized approach, RT-PCR was also performed, as well as a nasopharyngeal aspirate. Comparison: The number of patients needing ICU department, as well as longer hospital length of stay (LOS) was compared between the two groups. Outcome: To evaluate if there is a connection between the circulating VD status at the start of the illness and the severity among infants diagnosed with bronchiolitis.	Compared to those with VD values of ≥30 ng/mL, children with <20 ng/mL had a greater probability of needing ICU and a longer LOS. Patients at the lowest end of circulating 25(OH)D concentration had longer LOS than those with the highest tertile, but neither unadjusted nor adjusted models found a relationship between the free 25-OHD concentration and the clinical outcome of bronchiolitis.	This research concluded that values of <20 ng/mL put infants at risk of needing intensive care and longer hospital days; thus, this vitamin plays an essential role in the outcome of bronchiolitis. The observed correlation between total 25(OH)D concentration and bronchiolitis severity, rather than bioavailable or free 25(OH)D, shows that total concentration may be a more accurate indicator of ARTI.	YES
Toivonen et al. [57]	Multicenter prospective cohort study	Participants: In total, 1005 infants under 12 months hospitalized with bronchiolitis were distributed into two groups. The lower serum concentration of 25OHD included 498 patients, and the higher serum concentration had 507 patients. Intervention: In the first 24 h of hospital admission, staff collected blood and nasopharyngeal airway samples. Comparison: The lower vitamin D level group (<26.5 ng/mL) and the higher one (≥26.5 ng/mL) were compared against the days of ICU stay. Outcome: To analyze the possible 25(OH)D—ICU admission relation and use of mechanical ventilation (such as CPAP or intubation) during hospitalization.	25(OH)D and nasopharyngeal microbiota profiles appear to be related to the risk of needing intensive care, as patients at the lower spectrum of 25(OH)D levels had a higher risk of ICU (24.5%) when it comes to Hemophilus when compared with the higher-level group (16.2%). In contrast, there were no significant associations among those with higher 25(OH)D concentrations.	The study concludes that circulating 25OHD concentrations are associated with certain nasopharyngeal metabolomic signatures, like increased pro-inflammatory lipids, which may function as mediators in the microbiome-host interactions in the airways.	YES
Hueniken et al. [58]	RCT	Participants: In total, 703 children between 1 and 5 years of age with upper respiratory tract infections (URTIs) were divided into a group that received a high-dose supplementation of vitamin D (349 children) and a group that received the standard dose (354 children). Intervention: Oral VD doses of 2000 IU/day or 400 IU/day were randomly administered to children in the study for one winter season. Comparison: The two groups were compared for the risk of developing URTIs. Outcome: To analyze the effects of vitamin D concentration of 2000 IU/day on outpatient or emergency department (ED) visits correlated with antibiotic prescriptions for URTI.	The incidence rate ratio of the high supplementation group did not reduce the severity of the URTIs symptoms (IRR = 0.97), together with the frequency of ED and outpatients’ visits (IRR = 1.17 and IRR = 1.16, respectively). The IRR of antibiotic prescriptions for the two groups had similar results, of only 1.02.	This research concluded that high doses of VD in wintertime did not lessen the severity of the symptoms in URTI compared to the normal dosage for children, as the American Academy of Pediatrics (AAP) advised. High-dose vitamin D supplementation also did not reduce the incidence of ED visits, outpatient doctor visits, or antibiotic prescriptions.	NO
Kun et al. [59]	Prospective cohort study	Participants: In total, 175 children with Mycoplasma pneumoniae (MPP) were divided into two groups according to the existence of bronchial mucus plugs (BMP). Thus, 73 children were included in a BMP group and 102 in a non-mucus group. Intervention: Fiberoptic bronchoscopy of the mucus secretions was performed on patients where it was blocking the respiratory tract, and for the rest of the children where they could not be easily removed, doctors used a cell brush or biopsy forceps. Comparison: The two groups of patients were compared by looking at 25(OH)D concentrations, IL8, and other inflammatory markers. Outcome: To evaluate any associations between these specific blood tests and clinical characteristics in the pediatric population with mycoplasma pneumonia (MPP).	25(OH)D, IL8, neutrophils and other inflammatory cells were lower in the non-BMP group when compared with the BMP group. On the other hand, the relation between the blood VD and the other inflammatory markers tested here appeared to be inversely related.	According to this research, vitamin D could be used as a marker in diagnosing children with MPP. Young patients with MPP often have vitamin D insufficiency, and its levels are strongly correlated with the presence of inflammatory cells and the severity of the illness. Children with MPP and BMPs had serum 25(OH)D concentrations lower than those without MPP and BMPs.	YES
Oktaria et al. [60]	Prospective cohort study	Participants: In total, 422 pregnant women and their subsequent babies were observed for ARTI episodes for a period of 12 months following delivery. Intervention: At birth, newborns were tested for their VD levels, and a follow-up occurred 6 months later. Comparison: Blood results of children’s vitamin D taken 6 months apart were analyzed against the incidence of ARTIs. Outcome: To observe the incidence of pneumonia concerning VDD for the 1st year of life.	Within the selected sample of patients, 1601 ARTIs incidents were found to have occurred over 1 year. In total, 96 and 7 pneumonia cases matched the WHO criteria for pneumonia and severe pneumonia, respectively (8.7% of the total). Nearly all newborns (96%) had at least one instance of ARI that was not pneumonia. The 9 to 12 months age group had the greatest pneumonia frequency, whereas newborns under 3 months had the lowest. For non-pneumonia and pneumonia illnesses, the median length of full symptom resolution was 7 days and 15 days. Between those with VDD and non-VDD at birth, there was no difference in the prevalence of the 1st episode of pneumonia when compared with the 2nd one within 6 months, as well as other incidences of pneumonia from 6 to 12 months. It appears as if these events were unrelated to vitamin D status at 6 months of age.	VD status of newborns, influenced by the mother’s exposure to the sun, influenced very little the occurrence rate of pneumonia in the first 6 months of life. Still, the overall frequency rate was not considerably changed by the status of vitamin D (whether at birth of after 6 months). Reducing VDD at birth by giving supplements to mothers or increased sun exposure during pregnancy has very little potential to reduce ARTIs.	NO
Oktaria et al. [61]	Cross-sectional study	Participants: In total, 127 hospitalized patients diagnosed with pneumonia (2 to 59 months old) whose vitamin D concentrations were measured. The study sample was divided between those who had VDI or VDD (25 children), and those whose levels were normal (102 children). Intervention: Doctors measured the total VD2 and VD3 using liquid chromatography [tandem mass spectrometry. Comparison: The two groups were analyzed, comparing the vitamin D concentration and their risk of developing pneumonia. Outcome: To observe if there is any correlation linking vitamin D and symptom severity in these patients.	Infants younger than 6 months needed longer LOS in the hospital (more than 5 days). However, this seemed to be more related to the low birth weight and poor nutritional status on admission, which were also risk factors for hypoxemia.	1/5 children in this study group were VDD. Still, the authors concluded that this status was not associated with the severity of pneumonia, and with the presence of other danger signs, such as hypoxemia or the quantity of oxygen required. Also, the 25(OH)D concentration was unrelated to the hospitalization duration.	NO
Akeredolu et al. [62]	Cross-sectional study	Participants: In total, 270 children were put into a group for those with pneumonia (135), and another one with 135 apparently healthy children. The children were aged 1 to 59 months. Intervention: Vitamin D serum levels were measured for both groups. Comparison: The two groups were compared for their vitamin D concentration. Outcome: To assess the suitable intervention of vitamin D supplementation in preventing acute pneumonia in children younger than 5 years of age.	Statistics in this study showed a difference between the means of the serum concentration (52.14 vs. 60.91) for the 1st group when compared to the control group. Also, most children in the pneumonia group (91.1%) were VDI (with VD concentration of less than 75.0 nmol/L) when compared to the healthy children, that were almost 20% less (71.9%).	This study supports the link between vitamin D and acute pneumonia in pediatric population. To lower children’s risk of pneumonia, the authors advise that techniques and treatments, including supplementation of VD, better nutrition (food that includes vitamin D), and additional measures regarding increasing children’s vitamin D status should be seriously examined.	YES
Bayramoğlu et al. [63]	RCT	Participants: In total, 103 COVID-19-positive children between 1 and 18 years of age were put into 3 groups: asymptomatic patients (29); patients with mild infections (40); and those with severe infections (34). Patients in the last group had pneumonia confirmed by physical examination and X-ray/CT Intervention: The infection with the virus was confirmed by PCR, with sample collected through a nasopharyngeal swab. Comparison: The 3 groups (asymptomatic, mild, and severe) were compared against their vitamin D concentration. Outcome: To assess the symptoms severity and inflammatory markers in the pediatric population during COVID-19 concerning the VD status.	Compared to the mild and asymptomatic groups, the 3rd group showed lower lymphocyte counts and significantly C reactive protein, lymphocyte count, pro-calcitonin, fibrinogen, and D-dimers, which are signs of inflammation. Some children were also VDI, and the ratio was 70.6% in the severe group. The VD level had a positive relation with the lymphocyte count (r = 0.375), and an inverse association with age (r = −0.496), CRP (r = −0.309), and fibrinogen levels (r = −0.381).	This study revealed an association between insufficient concentrations of 25(OH)D and the evolution of the COVID-19 infection. The 3rd group had worse vitamin D levels and higher inflammatory cells. Although vitamin D’s effects on the immune system are important as the evidence suggests that appropriate 25OH vitamin D concentration assists the body’s ability to fight off viral and bacterial infections and minimize inflammation.	YES
Doğan et al. [64]	Prospective cohort study	Participants: In total, 176 children aged 1–18 years divided into two groups: one with 88 children with COVID-19 disease and the other with 88 healthy children. Intervention: Electrochemiluminescence immunoassay was used to measure the serum VD. Comparison: The 25(OH)D concentrations of the groups were compared in relation to the severity of the infection. Outcome: To evaluate if there is a connection between the severity of the COVID-19 infection and this vitamin.	When looking at the two sets of children, COVID-19-positive patients’ mean blood 25 hydroxy cholecalciferol levels were lower (11.73) than those of the other group (18.14). Also, the study shows a significant link between VD and Zn values in the two groups (r = 0.245).	Since the two assessment markers (vitamin D and Zn) showed a connection with the infection, being higher in healthy children, the authors recommend supplementing of the two as a supportive treatment for COVID-19 infection.	YES
Jaybhaye et al. [65]	RCT	Participants: In total, 108 children with RRIs (both inpatients or outpatients) aged from six months to 15 years in one group and 55 healthy children of the same age group who visited the hospital throughout the research period for immunizations and regular checkups, as the control group. Intervention: The serum 25-hydroxyvitamin D collected from venous blood from patients and controls was analyzed. Comparison: The intervention group was compared with the control one Outcome: To evaluate whether the RRIs and 25 hydroxyvitamin D are connected.	Essentially, all children with RRIs had less than normal vitamin D levels. Around 75% of these patients had VDI, and the rest had VDD. With a *p*-value of less than 0.001, these variations in 25(OH)D status of the two groups were statistically significant.	Comparatively, to controls, children with recurrent respiratory infections had a significantly high rate of vitamin D insufficiency. Children with recurrent respiratory infections should be managed with an evaluation of their vitamin D level.	YES

**Table 2:** Results of 6 meta-analyses included in the study regarding the effectiveness of administering vitamin D supplements.

Study	Study Design	Pico Framework	Results of the Study	Conclusion	VIT. D Supplementation
Cariolou et al. [66]	Systematic review and meta-analysis of observational studies	Participants: In total, 52 studies that included 7434 children from 15 countries hospitalized with acute or critical conditions (sepsis and respiratory tract infections). Intervention: Serum level of vitamin D was analyzed against the RTIs. Comparison: The patients were either with a VDI status (<50 nmol/L) (3473, which accounts for 47.0%) or with a sufficient VD status. Outcome: To evaluate the prevalence of VDD in children with acute or critical conditions.	Of the total number of children in the study, almost half (47%) were VDD with less than 50 nmol/L, with a joint incidence rate estimation of 54.6% (48.5% to 60.6%). After ignoring studies with a small number of patients, the authors found out that the frequency of infections was comparable to the above findings, with 51.5%.	As 2463 young patients were at risk of mortality, this review (that included 18 cohort studies) revealed that this probability was linked with VDD status. Thus, a lower mortality rate can be achieved if children with VDD receive supplementation doses to normalize their VD level.	YES
Hong et al. [67]	Prospective birth cohort study	Participants: In total, 2244 infants monitored from birth to 6 months of age divided into four groups according to the frequency of VD supplementation Intervention: For the first 6 months of life, infants received every day a 400 to 600 IU dose of 25(OH)D. Comparison: The frequency of supplementation was from 0 to 5 to 7 days per week. Outcome: To assess the first episode of pediatrician-diagnosed RTI, or to determine if, in the first 6 months, children had no RTI events.	Infants without vitamin D supplements had their first RTI episode on average 60 days after birth, whereas those receiving supplements had it when they were older than 6 months. According to the authors, the risk ratios in the supplementing group that took supplements 5–7 days per week were 0.46 and 0.18, respectively, showing an inverse association between supplementation periods of time and the risk for various respiratory tract infections that would need hospitalization.	Infants who received vitamin D supplements in the first six months of life saw the onset of the first RTI later than those who did not. For all children without supplements, the median time until their first RTI was 60 days. For exclusively breastfed newborns, it was 36 days and for formula-fed infants it was 90 days.	YES
Pham et al. [68]	Systematic review and meta-analysis of observational studies	Participants: In total, 14 studies were included in a meta-analysis evaluating the ARTIs risk, and a further 5 were analyzed for severity of symptoms. Intervention: Blood 25 hydroxycholecalciferol was measured. Comparison: Blood levels were compared with the risk of developing RTIs. Outcome: To assess the risk and severity of ARTI in children with VDI or VDD.	When comparing the lowest to the highest 25(OH)D category, the pooled odds ratios were 1.83 and 2.46, respectively, showing an inverse relationship between 25(OH)D and risk and development of ARTIs. The likelihood of infections rose by 1.02 for every 10 nmol/L of 25(OH)D drop in serum. This was a straightforward trend, as and the risk of ARTIs increased most dramatically when the VD concentration was below 37.5 nmol/L.	According to the research, vitamin D significantly reduces the likelihood and severity of ARTIs, particularly in those with low concentrations. Still, finding the ideal concentration or the level below which supplementation would be beneficial proved difficult.	YES
Anitua et al. [69]	Systematic review and meta-analysis of observational studies	Participants: In total, 65 studies were deemed eligible, with a total of 50,554 participants. Intervention: 25(OH)D was determined from blood samples Comparison: The results were compared with the risk of developing RTIs. Outcome: To assess the risk and severity of ARTI in children with VDI or VDD.	Many studies stated that vitamin D supplementation reduced the risk of respiratory infections among all participants. Consequently, the protective effect of vitamin D was only substantial when taken regularly.	According to incidence count data, this meta- concluded that the incidence of upper and lower respiratory tract infections was noticeably decreased by vitamin D prevention.	YES
Kumar et al. [70]	Systematic review and meta-analysis of RCTs	Participants: In total, 18 RCTs totaling 1579 participants were included in this study. Intervention: Supplementing with vitamin D in addition to the treatment for asthma Comparison: Placebo groups were compared to the intervention groups Outcome: To assess the risks and severity of asthma in children in relation to their vitamin D status.	This meta-analysis included randomized control trials with VDI children whose levels were below 20 nmol/L. However, information on asthma aggravation was only given by one research, where vitamin D supplementation had no detectable impact on any of the outcomes that were reported. Other results did not significantly alter in any way. Using the fixed-effect model, the authors conducted a sensitivity analysis for results with heterogeneity of less than 50%. The conclusion was that these groups had no difference in any of the outcomes.	Vitamin D supplementation had no noticeable impact on asthma episodes needing systemic corticosteroids, according to the collective RCTs included in this research. Furthermore, there was no obvious change in the proportion of children experiencing asthma episodes. Therefore, vitamin D does not lessen the need for hospital stays and urgent care visits. There is little evidence to support the idea that vitamin D supplementation may be protective in children with asthma.	NO
Fang et al. [71]	Systematic review and meta-analysis of observational studies	Participants: Four trials totaling 13.367 participants examined the effectiveness of vitamin D in avoiding ARTIs. Intervention: The concentration of vitamin D was determined. Comparison: Infection rates between the VD groups and control ones. Outcome: To find out if there is any association between a higher dose of vitamin D and the incidence and outcome of ARTIs in children.	With 1.596 participants, two studies investigated the effectiveness of higher vs. regular supplementation doses of 25(OH)D when it comes to preventing ARTIs. There were few obvious differences between the two groups, and the included trials lacked substantial heterogeneity. Because the influenza A infection was reported in many studies utilizing different treatment arms, only that illness could be the subject of a meta-analysis. Compared to the lower dose of the VD group, the higher one had some decline in influenza A rates.	Investigating the effectiveness of vitamin D supplementation in preventing these illnesses produced mixed findings. According to this research, vitamin D administration offers no noticeable advantage over a placebo in terms of considerably lowering ARTI rates, but it did show some improvement when it came to the Influenza A virus. Furthermore, a high amount of vitamin D had no advantages over administering the recommended quantity. Additionally, in most infections recorded, the frequencies of various viral infections were equally independent of the regimen or organism that caused the illness.	NO

**Table 3:** US National Institute of Health recommended amount of vitamin D [96].

Life Stage	Recommended Amount
<12 months	10 mcg (400 IU)
Children 1 to 13 years	15 mcg (600 IU)
Teens 14 to18 years	
Adults 19 to 70 years	
Adults ≥71 years	20 mcg (800 IU)
Pregnant and breastfeeding women	15 mcg (600 IU)

## Data Availability

No new data was created, as this research was based on secondary data.
